# Six-degree-of-freedom knee motion during treadmill walking in mechanically and kinematically aligned TKA

**DOI:** 10.1038/s41598-026-52076-8

**Published:** 2026-05-14

**Authors:** Ann-Kathrin Einfeldt, Lars Tücking, Peter Savov, Max Ettinger, Henning Windhagen, Eike Jakubowitz

**Affiliations:** 1 Laboratory for Biomechanics and Biomaterials, Department of Orthopaedic Surgery, Hannover Medical School, Anna-von-Borries-Str. 1-7, 30625 Hannover, Germany; 2 Department of Orthopedic Surgery, DIAKOVERE Annastift, Hannover Medical School, Anna-von-Borries-Str. 1-7, 30625 Hannover, Germany; 3 Department of Orthopaedic Surgery, Pius Hospital, University of Oldenburg, Georgstraße 12, 26121 Oldenburg, Germany

**Keywords:** Kinematic Alignment, Mechanical Alignment, CPAK Classification, Range of Motion, Patient-Specific Alignment., Diseases, Health care, Medical research

## Abstract

The current literature remains inconclusive about whether kinematic alignment (KA) is superior to mechanical alignment (MA) in total knee arthroplasty (TKA). Therefore, this randomized controlled trial sought to provide further clarification. 39 MA patients, 33 KA patients, and 9 healthy controls were included. All participants underwent three-dimensional gait analysis on a treadmill one day before surgery and one year postoperatively. A preoperative range of motion (ROM) score was calculated and correlated with the postoperative Forgotten Joint Score (FJS). One year after surgery, no significant kinematic differences were observed between MA and KA. However, in Coronal Plane Alignment of the Knee (CPAK) type 1 patients, KA knees more closely reproduced the gait of healthy controls and showed higher walking speed. Correlation analysis revealed that patients with a higher preoperative ROM achieved better outcomes with KA, whereas female patients with lower ROM benefit more from MA. Although no kinematic differences were detected, the strong trend toward reduced joint space in MA compared to KA in CPAK type 1 patients may result from subtle deviations in joint-line restoration or postoperative soft-tissue tension. Both factors can affect load distribution and joint perception. The present findings support a patient-specific approach to alignment selection in TKA.

**Supplementary Information:**

The online version contains supplementary material available at 10.1038/s41598-026-52076-8.

## Introduction

Total knee arthroplasty (TKA) is one of the most commonly performed orthopaedic procedures worldwide. Approximately 720,000 TKAs were performed in Europe in 2019^1^. In Germany, 170,494 TKAs were performed in 2018, and these numbers are expected to increase to 225,957 by 2050 due to the aging population and rising prevalence of osteoarthritis^2^.

Historically, dissatisfaction rates among TKA patients have consistently been reported at 20% or higher^3–5^. As a result, along with technical advancements in implant design and surgery, including computer-assisted navigation and robotic technology, new alignment philosophies have been introduced to improve outcomes. One such philosophy is kinematic alignment (KA), which was introduced as an alternative to the traditional mechanical alignment (MA). While MA seeks to position the prosthesis along the neutral mechanical leg axis, KA aims to restore the patient’s individual, constitutional limb alignment rather than enforcing neutral alignment^6^. Consequently, a certain degree of varus alignment may be preserved in patients with constitutionally varus knees, although preoperative alignment does not necessarily reflect the native alignment due to disease-related progression.

A recent review, focusing on studies published from 2010 onwards, reported a decrease in the average rate of dissatisfaction among TKA patients to approximately 10%^7^. This improvement is likely attributable to multiple factors, including advances in surgical techniques, evolving alignment philosophies, and innovations in implant design. However, due to the considerable heterogeneity among the included studies in terms of methodology and patient populations, the specific factors contributing to this reduction remain unclear. Another recent review comparing patient satisfaction between MA and KA patients found that both groups reported high levels of postoperative satisfaction, with no statistically significant differences between them^8^. The authors criticize the low quality of the included studies and the inability to control for confounding factors, such as implant type and postoperative rehabilitation, highlighting the need for further research in this area.

Given the rising prevalence of osteoarthritis and the increasing focus on alignment techniques, it is important not only to assess patient satisfaction but also to explore how different surgical philosophies affect patient-reported outcome measures (PROM) following TKA. When examining the current literature comparing outcomes between MA versus KA patients, many reviews report a slight tendency toward better PROM in KA patients^9–12^, whereas others find no significant differences between the groups^13–15^. A recent umbrella review published in 2024 concluded that the available evidence remains insufficient to determine whether KA is superior to MA^16^. The authors noted that most of the included reviews exhibited methodological limitations in their meta-analysis, potentially leading to biased results. These limitations included inconsistent time points of the analysis, duplicate inclusion of patients, inclusion of studies with incorrect definitions of alignment techniques or those using restricted kinematic alignment, and inappropriate pooling of bilateral and unilateral TKA studies.

Another reason why large differences between groups are often not observed is that many PROMs are influenced by ceiling effects when both patient cohorts achieve high functional levels^17^. Therefore, objective functional outcomes should also be assessed during activities of daily living to determine whether one alignment strategy yields superior performance. There is a paucity of studies evaluating functional outcomes of MA and KA patients using gait analysis. Two investigations examined plantar pressure distribution during gait and reported a more physiological loading pattern in KA patients compared to MA patients^18,19^. Five studies have conducted 3D gait analysis comparing mechanically and kinematically aligned TKA. Three of these reported no significant differences between the two strategies^20–22^. One study demonstrated that MA patients showed significant deviations from the control group, whereas KA patients more closely reproduced the gait patterns of healthy controls^23^. Another study investigated joint kinetics and identified a significantly higher knee adduction moment (KAM) in MA compared to KA patients^24^. In contrast, the study by Bauer et al.^20^ reported a smaller KAM in MA patients than in KA patients, and the study by McNair et al.^21^ did not find any significant differences between these groups. Most studies included relatively small sample sizes, ranging from 10 to 21 knees per group. None of these investigations assessed six degrees of freedom (6-DOF) knee kinematics in MA and KA patients. As the knee joint is a roll-glide joint, it is important to investigate not only rotary movements, but also translations. There might be important kinematic differences between these two alignment philosophies that could not be addressed until now, and these differences may be grounded in translational motions. Therefore, the aim of the present randomized, observer-blinded, prospective study was to analyze the complete 6-DOF knee joint kinematics following TKA using the MA and KA approaches. A non-osteoarthritic cohort served as a reference group. We hypothesize that, compared with healthy controls, KA patients will demonstrate smaller deviations in knee kinematics during level and downhill walking than MA patients one year postoperatively.

## Materials and methods

A single-center, double-blinded, randomized controlled trial was conducted. The sample size was determined using G*Power 3.1.5 based on the primary outcome of no difference in the Forgotten Joint Score (FJS) one year postoperatively (α = 0.05 and power = 0.8). This analysis yielded a required sample size of at least 46 patients per group. The clinical study was registered at the german registry for clinical studies with the number DRKS00024567 on 14.04.2021 and approved by the local ethics committee (Ethics Committee of the Hannover Medical School, #7495). All methods were performed in accordance with the relevant guidelines and regulations according to the Declaration of Helsinki. Following informed written consent, 98 patients (three additional per group) who received the GMK Sphere^®^ implant (Medacta International, Castel San Pietro, Switzerland), and nine healthy controls (18 legs) were enrolled in the study. Inclusion criteria comprised patients with symptomatic knee osteoarthritis and a medial proximal tibial angle (mPTA) between 85° and 90°. The exclusion criteria are summarized in Table 1. Patients were randomly assigned to one of the two study groups and remained blinded until the study was completed. Gait laboratory staff were likewise blinded to group allocation until after the one-year follow-up assessment.

**Table 1:** Exclusion criteria.

Exclusion criteria
Pregnancy or breastfeeding
Difference in the radius of medial and lateral condyles > 2 mm
Previous osteotomy around the knee
Body mass index (BMI) > 30 kg/m^2^
Ligament instability likely to require higher level of constraint
Previous infection or inflammatory disease
Any patient who cannot or will not provide informed consent for participation in the study

Surgical procedures were performed using patient-specific instrument blocks (PSI, MyKnee^®^, Medacta International) and a uniform medial-pivot implant design to ensure accurate execution of the planned alignment philosophy (MA vs. KA). In the MA group, component positioning was planned with a mPTA and lateral distal femoral angle (lDFA) of 90°. In contrast, in the KA group, component alignment was adapted to the native anatomy to restore the femoral and tibial joint lines as closely as possible to their pre-osteoarthritic condition. For study inclusion, restriction thresholds were applied for mPTA and lDFA, allowing a maximum deviation of 5° from neutral alignment.

All participants underwent 3D treadmill gait analysis (h/p/Cosmos Holding GmbH, Nußdorf¸ Deutschland) on both a level surface (0%) and a downhill slope (12%, Illustration of downhill setup in S1) at their maximum achievable walking speed. In accordance with Wiik et al.^25^, treadmill speed was increased incrementally until the participant reached maximum walking speed, following a 2-minute acclimatization period at 4 km/h, if possible. Otherwise, the acclimatization period was performed at maximum possible walking speed. Measurements were performed one day prior to surgery and one year postoperatively. Kinematic data were recorded using a 200 Hz motion capture system (Vicon Motion Systems Ltd., Oxford, UK) equipped with twelve MX infrared cameras. The system tracked the trajectories of 30 retroreflective markers (14 mm in diameter) placed on anatomical landmarks, according to the recently published MiKneeSoTA method^26^. This method minimizes motion artefacts caused by soft-tissue movements around the knee – one of the main limitations in gait analysis – and enables the acquisition of all six degrees of freedom (6-DOF) of the knee joint. To ensure consistent reference frames across measurements, the REFRAME algorithm^27,28^ was applied to all datasets. For abduction/adduction and internal/external rotation, the root mean square error (RMSE) was minimized using a weight of 1, whereas flexion was optimized towards the raw flexion with a weighting factor of 0.2. Rotations of the tibial reference frame around the flexion axis were fixed. Translations were not optimized but adapted using the rotational frame transformations. Joint rotations are presented as the rotation of the tibia relative to a fixed femur, whereas translations are expressed as the movement of the femur relative to a fixed tibia.

In addition, the FJS was obtained. Prior to statistical analysis, all data were tested for normality using the Lilliefors-Test. A two-tailed t-test was used to compare FJS values between groups, with a significance level of *p* = 0.05. Anthropometric parameters and gait speed were analyzed using one-way ANOVA followed by Bonferroni-corrected post hoc testing. For the evaluation of kinematic timeseries data, Statistical Parametric Mapping (SPM) was performed using ANOVA with Bonferroni post hoc analysis, with the critical significance threshold set at *p* = 0.02.

For further analysis of range of motion (ROM), the 3D minimum and maximum values for each of the 6 DOF were calculated. The absolute difference between these values defined the ROM score for each degree of freedom. Each ROM score was then normalized to a scale from one and 100 for each patient, where one represented the smallest and 100 the largest ROM score across all participants. The six normalized ROM scores were subsequently averaged to obtain a composite ROM_sum score between one and 100 for each patient. This preoperative ROM_sum score was then correlated with the postoperative FJS. All statistical analyses were performed using MATLAB (version 2024b, The MathWorks, Inc., Natick, USA).

**Fig. 1:**
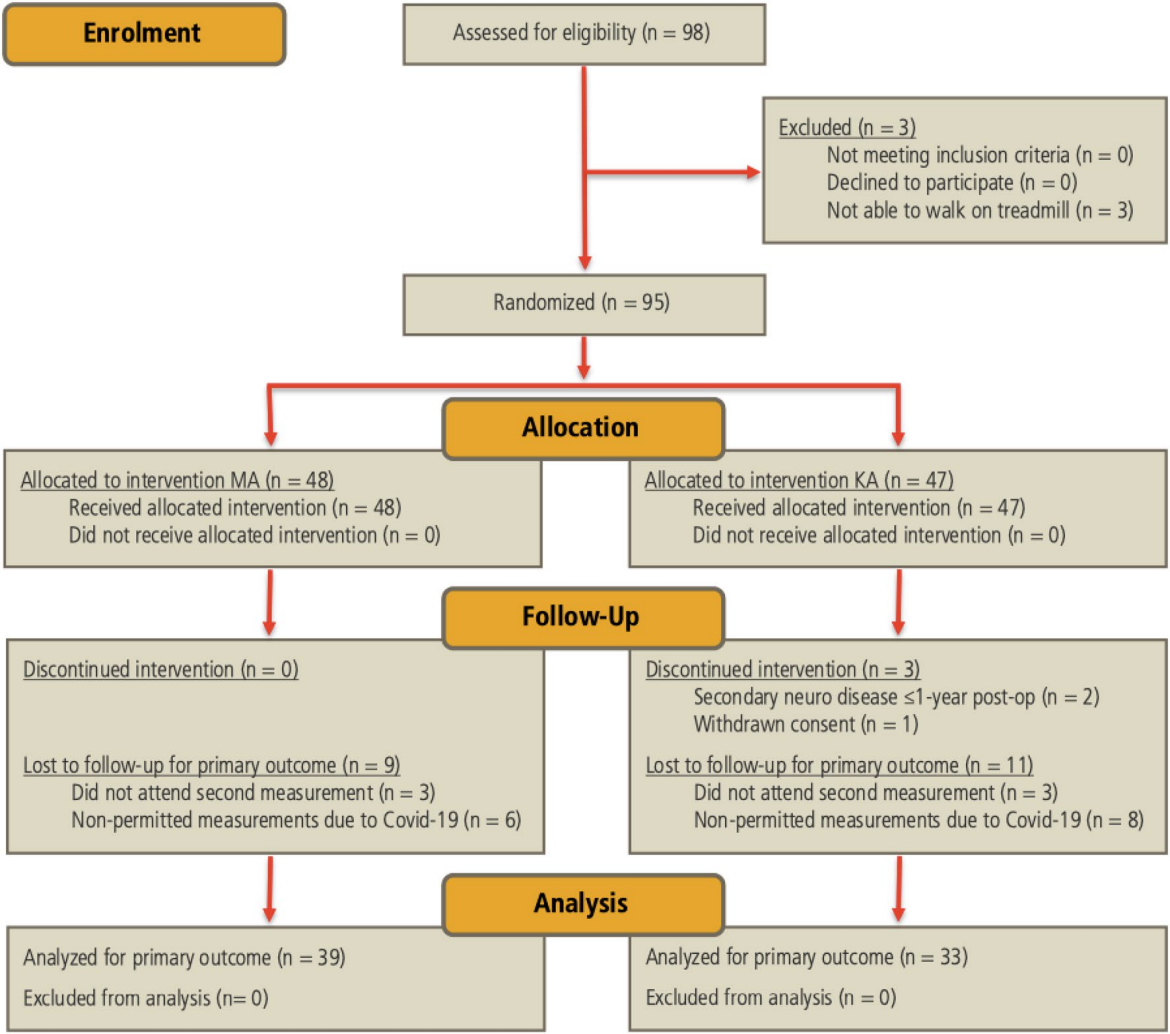
Flow chart of the double-blinded, randomized controlled trial.

## Results

Of the initially enrolled 98 patients, 26 were lost to follow-up, mainly due to COVID-19-related restrictions that temporarily prevented gait lab measurements (Fig. 1). The final cohort comprised 39 MA and 33 KA patients.

Pre- and postoperative anthropometric measurements revealed no significant differences between MA and KA patients, except for the preoperative BMI. The control group was significantly younger than both patient groups and had a lower BMI than the MA group (Table 2).

**Table 2:** Anthropometric characteristics of the mechanical alignment (MA), kinematic alignment (KA) and control (C) groups. Values are presented as mean ± SD. Significant p-values (*p* < 0.05) are printed in bold.

Parameter	MA *n* = 39	KA *n* = 33	Control *n* = 9	MA vs. KA	MA vs. C	KA vs. C
Sex [f:m]	12:25	8:25	4:5			
Age [years]	63.7 ± 10.8	68.3 ± 10.1	41.3 ± 16.5	0.25	**< 0.01**	**< 0.01**
Height pre-op [m]	1.71 ± 0.10	1.73 ± 0.10	1.73 ± 0.09	1.00	1.00	1.00
Weight pre-op [kg]	87.8 ± 14.7	84.1 ± 16.6	76.3 ± 13,6	0.91	0.14	0.54
BMI pre-op	30.1 ± 3.4	27.9 ± 3.6	25.3 ± 2.6	**0.03**	**< 0.01**	0.14
Height post-op [m]	1.71 ± 0.10	1.73 ± 0.10	1.73 ± 0.09	1.00	1.00	1.00
Weight post-op [kg]	86.7 ± 14.9	84.2 ± 16.8	76.3 ± 13,6	1.00	0.22	0.54
BMI post-op	29.6 ± 3.5	27.9 ± 3.8	25.3 ± 2.6	0.13	**< 0.01**	0.16

### Gait analysis

**Fig. 2:**
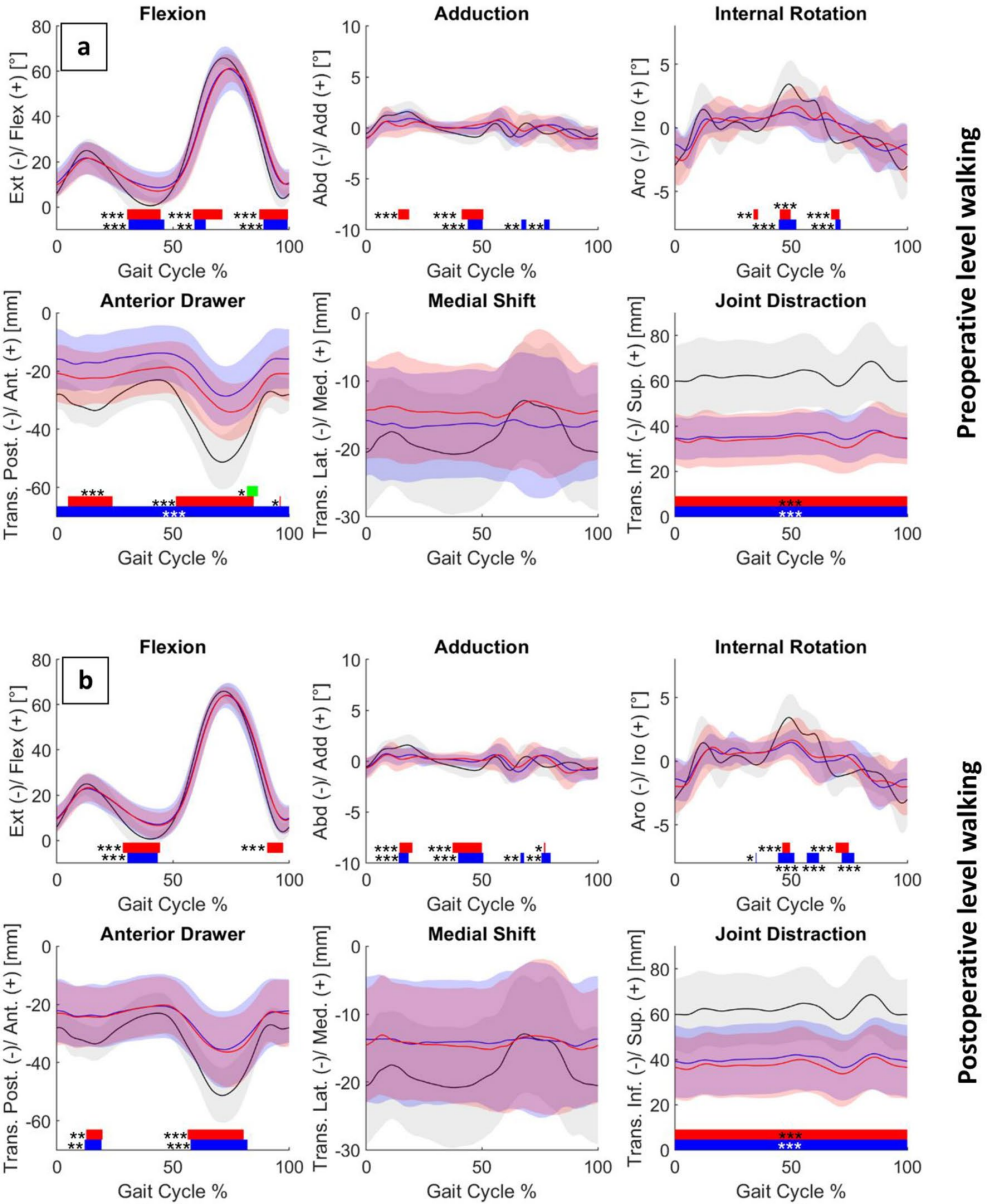
Mean ± SD (shaded areas) of pre-(a) and postoperative (b) level walking for MA (red), KA (blue) and control (black) groups. Joint rotations are shown in the upper row and translations in the lower row. Bars indicate significant differences between groups. red: MA vs. C, blue: KA vs. C, green MA vs. KA (*p=0.02; **p=0.01; *** *p* < 0.01).

Preoperatively, MA and KA patients did not differ significantly under either walking condition, except for a smaller posterior knee translation during the swing phase in the KA group (Fig. 2a; Supplementary Figure S2 for downhill walking). Compared with controls, both patient groups exhibited a reduced knee extension during terminal stance of around 6° in MA and 7° in KA patients (*p* < 0.01) and terminal swing (5° for both groups, *p* < 0.01), as well as reduced knee flexion of around 10° during initial swing (MA: *p* < 0.01; KA: *p* = 0.01). KA patients also showed a significantly smaller posterior knee translation of around 14 mm (*p* < 0.01) throughout the entire gait cycle, whereas in MA patients this reduction of around 9 mm was only significant during loading response and mid-swing (*p* < 0.01). In the frontal plane and for internal-external rotation, group differences occurred only at isolated time points during the gait cycle.

Inpatient groups demonstrated a significantly reduced joint space of around 26 mm in MA and 27 mm in KA patients throughout the entire gait cycle in the knee distraction graph (*p* < 0.01). Overall, the preoperative deviations from the control group were similar for both alignment groups. During downhill walking, both groups showed significantly reduced knee flexion of around 11° during the loading response (*p* < 0.01), but no reduction in knee extension during terminal stance. For all other motion directions, intergroup differences increased compared with the level-walking condition (Supplementary Figure S2).

Postoperatively, no significant differences were observed between MA and KA patients during either level or downhill walking (Fig. 2b for level walking and Supplementary Figure S2 for downhill walking). In the sagittal plane, deviations from the control group were smaller in KA patients, with only a residual reduction in knee extension of around 6° during terminal stance (*p* < 0.01) and reduced posterior knee translations during loading response (10 mm, *p* = 0.01) and mid-swing (17 mm, *p* < 0.01). In the frontal and transverse planes, differences from controls were slightly greater, particularly in KA patients. Both alignment groups continued to show significantly reduced knee joint distraction of around 23 mm in MA and 20 mm in KA patients throughout the entire gait cycle (*p* < 0.01). During downhill walking, similar results were observed, with slightly greater deviations from controls (Supplementary Figure S2).

**Fig. 3:**
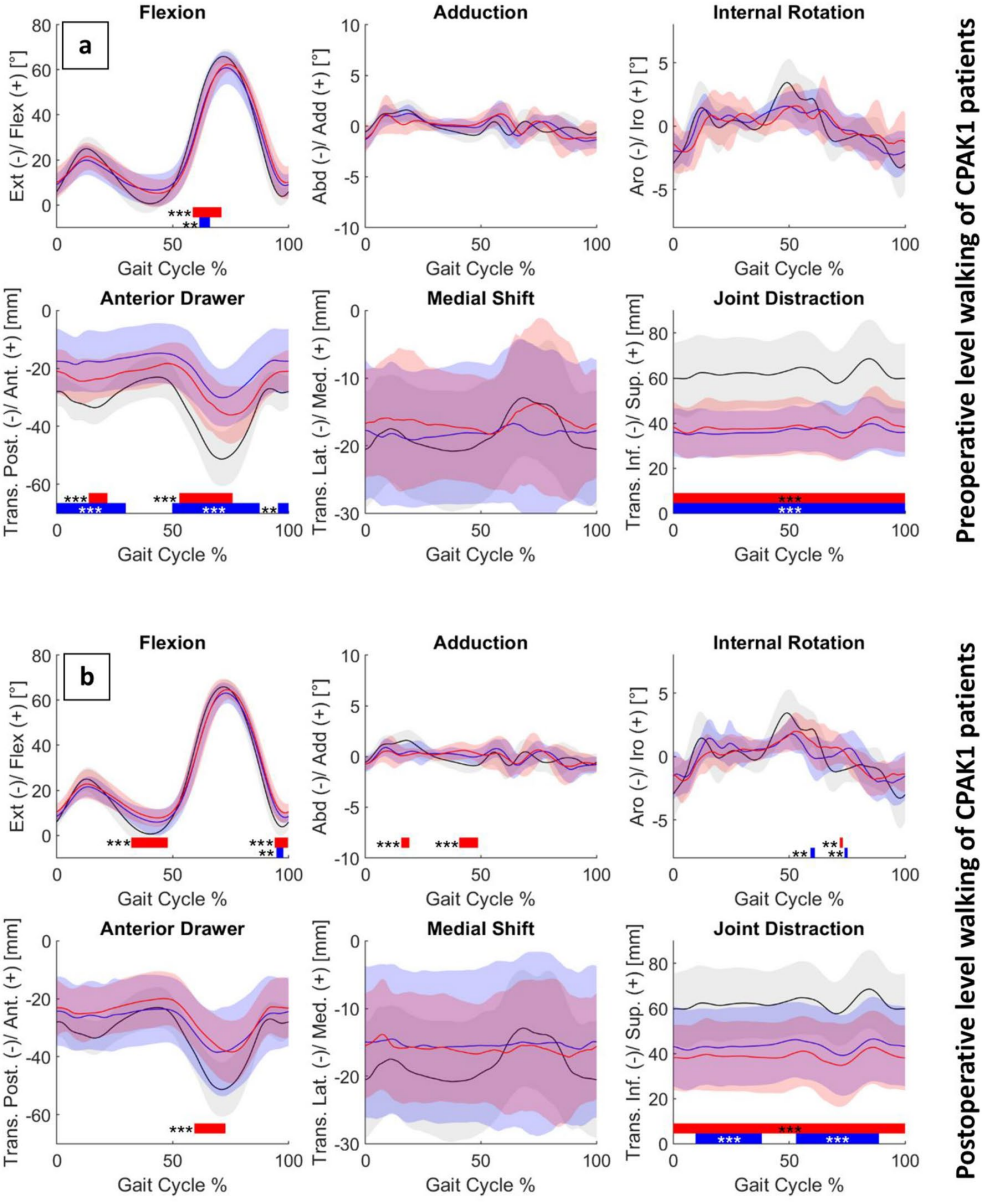
Mean ± SD (shaded areas) of pre-(a) and postoperative (b) level walking for CPAK1 MA (red), CPAK1 KA (blue) and control (black) groups. Joint rotations are shown in the upper row and translations in the lower row. Bars indicate significant differences between groups. Red: MA vs. C, blue: KA vs. C (*p=0.02; **p=0.01; *** *p* < 0.01).

When considering only a subgroup of CPAK type 1 patients – those with preoperative varus legs^29^– no significant differences were found between MA and KA patients either pre- or postoperatively (Fig. 3a, b). Compared with controls, both groups exhibited significantly reduced knee flexion of around 9° during early swing phase preoperatively (MA: *p* < 0.01; KA: *p* = 0.01) (Fig. 2a). KA patients showed reduced posterior knee translation of around 17 to 20 mm throughout the first half of the stance phase (*p* < 0.01) and at two timepoints in swing phase (*p* < 0.01 and *p* = 0.01), whereas in MA patients this reduction was limited to the loading response (10 mm, *p* < 0.01) and early swing phase (16 mm, *p* < 0.01). Both groups demonstrated significantly reduced knee joint distraction of around 25 mm for KA and 14 mm for MA patients across the entire gait cycle (*p* < 0.01). During downhill walking, KA patients exhibited greater deviations from controls, particularly in rotational knee kinematics, whereas differences in knee translation were comparable to those observed during level walking for both groups (Supplementary Figure S3).

In the sagittal plane, both alignment groups showed a reduced knee extension during terminal swing postoperatively (MA: *p* < 0.01; KA: *p* = 0.01) (Fig. 3b). MA patients additionally demonstrated a reduced knee extension of around 7° during terminal stance (*p* < 0.01) and a reduced posterior knee translation of around 14 mm at the beginning of the swing phase (*p* < 0.01). In the frontal plane, MA patients exhibited significant deviations from controls during the stance phase (*p* < 0.01), whereas in the transverse plane, KA patients showed differences during the swing phase (*p* = 0.01). MA patients demonstrated a consistently smaller joint distraction of around 23 mm throughout the entire gait cycle (*p* < 0.01), while in KA patients this reduction (19 mm) was evident for approximately 70% of the gait cycle (*p* < 0.01). During downhill walking, KA patients exhibited greater deviations from controls in knee rotational kinematics, whereas knee translational deviations were more pronounced in MA patients (Supplementary Figure S3).

**Table 3:** Comparison of pre- and postoperative gait speeds of MA, KA, and control groups (top), and CPAK type 1 MA, and CPAK type 1 KA, and control groups (bottom) with corresponding p-values. Significance level was set to p = 0.05.

All		speed (m/s)			*p*-value		
	*slope*	MA *n* = 39	KA *n* = 33	Control *n* = 9	*MA vs. KA*	*MA vs. C*	*KA vs. C*
Pre-op	0%	1.06 ± 0.47	1.14 ± 0.41	1.87 ± 0.10	1.00	**< 0.01**	**< 0.01**
	12%	0.97 ± 0.33	1.05 ± 0.43	1.81 ± 0.10	1.00	**< 0.01**	**< 0.01**
Post-op	0%	1.36 ± 0.36	1.38 ± 0.42	1.87 ± 0.10	1.00	**< 0.01**	**< 0.01**
	12%	1.28 ± 0.36	1.31 ± 0.42	1.81 ± 0.10	1.00	**< 0.01**	**< 0.01**
**CPAK1**	*slope*	MA *n* = 13	KA *n* = 14	Control *n* = 9	*MA vs. KA*	*MA vs. C*	*KA vs. C*
Pre-op	0%	1.22 ± 0.40	1.34 ± 0.38	1.87 ± 0.10	1.00	**< 0.01**	**< 0.01**
	12%	1.24 ± 0.39	1.24 ± 0.39	1.81 ± 0.10	1.00	**< 0.01**	**< 0.01**
Post-op	0%	1.53 ± 0.35	1.53 ± 0.39	1.87 ± 0.10	0.63	**< 0.01**	0.06
	12%	1.43 ± 0.33	1.43 ± 0.41	1.81 ± 0.10	1.00	**< 0.01**	**0.04**

### Gait speed

Both pre- and postoperatively, the gait speed did not differ between MA and KA patients (Table 3). Compared with controls, both patient groups walked significantly slower during level and downhill walking in the preoperative condition (*p* < 0.01). Gait speed increased in all patients postoperatively. However, the differences from controls remained significant (*p* < 0.01), except for KA patients classified as CPAK type 1, who no longer showed significant differences postoperatively.

### Correlation with FJS

**Fig. 4:**
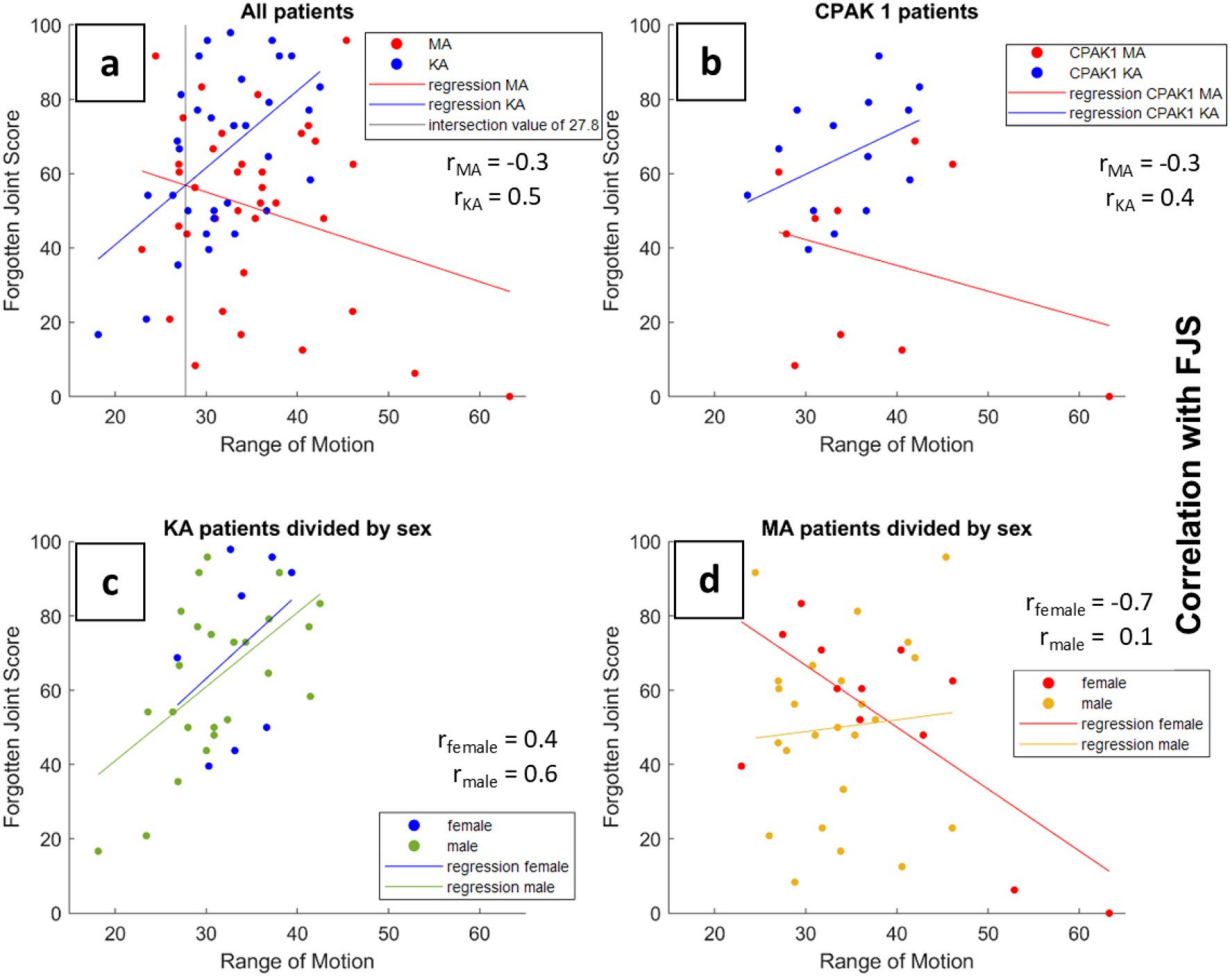
Correlation of preoprerative ROM with one-year postoperative FJS for all patients (**a**), for CPAK1 patients (**b**), for KAs divided by sex (**c**) and for MAs divided by sex (**d**).

One year postoperatively, the FJS differed significantly between MA and KA patients (MA: 51.5 ± 24.1; KA: 65.4 ± 21.7; *p* = 0.01). A similar significant difference was observed within the CPAK type 1 subgroup (MA: 44.1 ± 28.7; KA: 64.9 ± 16.0; *p* = 0.03). The correlation between the preoperative ROM_sum and the postoperative FJS revealed a moderate to strong positive relationship in KA patients (*r* = 0.5), whereas in MA patients a weak to moderate negative correlation was found (*r* = −0.3; Fig. 4a). When considering only CPAK type 1 patients, the positive correlation in the KA group decreased slightly (*r* = 0.4; Fig. 4b). After stratification by sex, a strong positive correlation was observed in male KA patients (*r* = 0.6) and a moderate positive correlation in female KA patients (*r* = 0.4; Fig. 4c). In contrast, among MA patients, males showed a weak positive correlation (*r* = 0.1), whereas females exhibited a strong negative correlation (*r* = −0.7; Fig. 4d).

## Discussion

This study found no significant differences in 6-DOF knee kinematics between MA and KA TKA one year after surgery. The advantages of KA, reflected in higher FJS scores, cannot be attributed to differences in 6-DOF knee kinematics, as these were comparable across the two alignment philosophies. These findings are consistent with those of Bauer et al., McNair et al. and Young et al.^20–22^, who also reported no significant kinematic differences between MA and KA patients during gait. The largest discrepancy between the two groups in the present study was observed in knee joint distraction, where KA patients exhibited slightly higher values, particularly among CPAK type 1 patients. As this effect remained constant throughout the gait cycle and followed a pattern similar to that of controls, it likely represents a difference in offset rather than a dynamic alteration in joint kinematics. A subsequent post hoc power analysis in G*Power (α = 0.05; power = 0.8) using the mean value over time and the corresponding standard deviation of the knee joint distraction (KA: 39.9 ± 15.2 mm; MA: 37.8 ± 13.8 mm) indicated that a sample size of 549 participants per group would be required to reach statistical significance. This suggests that the observed trend is most likely incidental. Among CPAK type 1 patients, however, the difference between the groups was larger, reducing the required sample size to 93 patients per group (KA: 43.4 ± 17.3 mm; MA: 37.2 ± 16.4 mm) to achieve statistical significance.

As reported by MacDessi et al.^29^, the majority of TKA patients are classified as CPAK type 2, representing a relatively neutral leg axis. This distribution was also observed in the present cohort, which may have influenced the overall results, as the biomechanical impact of alignment philosophy is expected to be smaller in this group. To address this, we additionally analyzed a subgroup of CPAK type 1 patients, i.e., those with preoperative varus alignment. Within this subgroup, KA patients exhibited smaller kinematic deviations from healthy controls compared to MA patients. Therefore, our initial hypothesis can only be confirmed for CPAK type 1 patients.

When examining postoperative frontal-plane rotations in CPAK type 1 patients, KA patients did not exhibit greater knee varus angles than MA patients during walking. This finding indicates that the static leg axis does not reflect the knee joint alignment during motion and that dynamic parameters may provide more meaningful insights when selecting or evaluating alignment techniques. Furthermore, KA patients in the CPAK type 1 group did not show significant differences in gait speed during level walking compared with controls, suggesting that the kinematic advantages may also be related to a more physiological gait speed. These observations align with the findings of Blakeney et al.^23^, who reported that KA patients walked faster and more closely reproduced the gait patterns of healthy controls than MA patients, although CPAK classification was not reported in their work. To further explore the relationship between gait speed and knee kinematics, we analyzed CPAK type 1 patients who were able to walk at 1.11 m/s both pre- and postoperatively (*n* = 10 per group). In this subgroup, KA patients again showed smaller deviations from controls (Supplementary Figure S4), suggesting that gait speed plays only a minor role in explaining the observed kinematic differences. These findings further support the hypothesis that CPAK type 1 patients are more likely to benefit from kinematic alignment. Other studies that stratified patients according to CPAK classification also reported better patient-reported outcomes in those who remained within their original CPAK class after TKA, compared to those whose classification changed postoperatively^30,31^. This underscores the importance of differentiating patient cohorts based on preoperative alignment characteristics and highlights the need to identify which patient subgroups may benefit most from each alignment philosophy, rather than pursuing a universal “optimal” technique.

To further investigate this relationship, we correlated preoperative ROM with postoperative FJS values. The results suggest that patients with higher preoperative ROM (ROM_sum > 27.8) tended to achieve better outcomes with the KA technique than with MA. As more patients had a greater ROM_sum than 27.8, more patients would have benefited from the KA surgical technique. This could explain why the current literature slightly favors KA, as it seems to be the better choice for most patients. Conversely, female patients with a lower preoperative ROM appeared to achieve better outcomes with MA. These findings highlight the potential of a patient-specific approach, which should be further refined by incorporating additional biomechanical parameters and clinical factors.

Due to the COVID-19 pandemic, the present study experienced substantial participant drop-outs, resulting in the initially calculated sample size of 46 patients per group not being achieved. Although the original power calculation was based on FJS scores and this study primarily focuses on gait parameters, it still, to our knowledge, represents one of the largest gait analysis cohorts comparing the MA and KA approaches for TKA. For example, Young et al.^22^ and Yeo et al.^32^ conducted gait analyses with only 10 participants per group, McNair et al.^21^ included 15 MA and 14 KA patients, and Blakeney et al.^23^ examined 18 patients per group. Only the recent study by Bauer et al.^20^ investigated a comparable number of participants. Despite randomization, MA patients in this study had a significantly higher preoperative BMI than KA patients. However, as no differences in preoperative kinematic outcomes were observed between the groups, BMI does not appear to have influenced gait kinematics in this cohort. The control group was significantly younger than both patient groups. To address potential age-related bias, we also compared the results to the unoperated contralateral legs of our patients and found minimal differences between groups. Nevertheless, as the degree of knee joint degeneration in these legs is unknown, interpretation of these comparisons remains limited. In our view, younger, healthy controls represent a more appropriate reference group, as degenerative effects on knee joint biomechanics can be excluded with certainty. We acknowledge that treadmill walking does not necessarily replicate real-world overground walking. However, we chose this setting because it is more consistent and allows us to capture more gait cycles, as well as downhill walking.

Furthermore, the current version of the ANOVA post hoc analysis implemented in SPM has not yet been formally validated; however, the developers report that potential errors are expected to be minimal^33^. It should also be noted that study inclusion criteria were restricted to patients with a maximum varus deformity of 5°. At the time of the study, ethical approval to include patients with more pronounced varus deformities was not granted. Future investigations should therefore include cohorts with greater preoperative varus alignment to improve generalizability.

## Conclusion

This randomized controlled trial did not reveal significant differences in knee kinematics between MA and KA during level and downhill walking one year after surgery. While most previous studies have reported similar findings, none have differentiated patients according to their CPAK classification. To our knowledge, this is the first study to analyze CPAK type 1 TKA patients separately and to demonstrate, that within this subgroup, KA patients more closely reproduced the gait pattern of healthy controls than MA patients. Moreover, the present study represents an initial step toward shifting the focus from a general debate over the “best” alignment philosophy to a more patient-specific approach – one that aims to identify which patient groups are most likely to benefit from each technique.

Although no kinematic differences were observed, the consistently reduced degree of joint distraction in TKA patients most likely reflects an offset shift rather than an actual change in kinematic knee pattern. The strong trend toward reduced joint space in MA compared to KA in CPAK type 1 patients may result from subtle deviations in joint-line restoration or postoperative soft-tissue tension. However, both are key factors influencing load distribution and joint perception. Recognition of this effect may be crucial for subsequent clinical and biomechanical work aimed at fully understanding how surgical alignment affects functional joint behavior.

## Electronic Supplementary Material

Below is the link to the electronic supplementary material.

Supplementary Material 1

Supplementary Material 2

## Data Availability

The datasets generated and analyzed during the current study are available from the corresponding author on reasonable request.
