# Supplementary material for: Six-degree-of-freedom knee motion during treadmill walking in mechanically and kinematically aligned TKA
Source: Sci Rep. 2026 May 14;16:15109. doi: 10.1038/s41598-026-52076-8 (PMC13172103; doi:10.1038/s41598-026-52076-8)
Supplement: Supplementary file 1 — Supplementary Material 1 [file 41598_2026_52076_MOESM1_ESM.pdf]

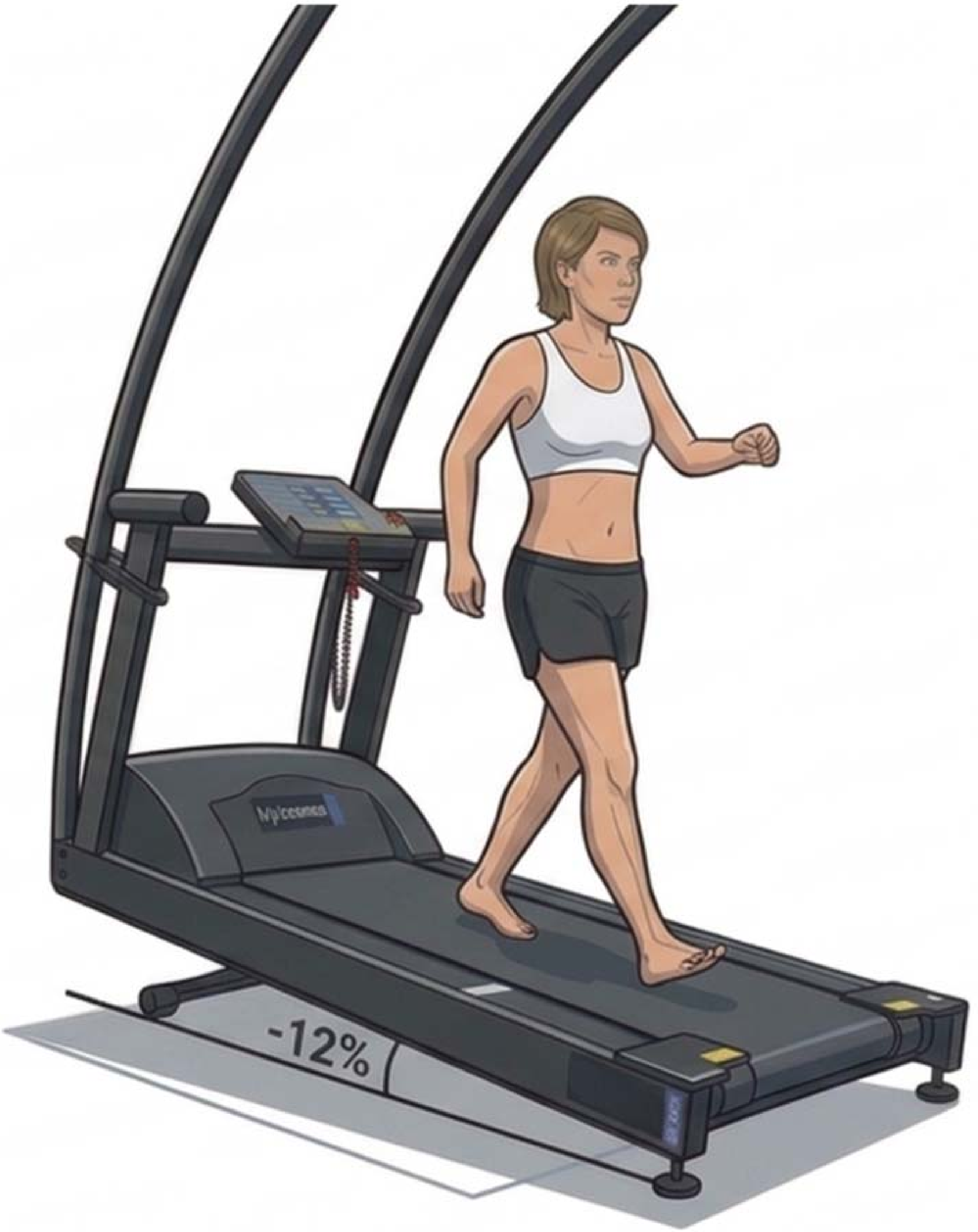

Supplementary Figure S1: Experimental 12% downhill walking setup using the treadmill (created with Nano Banana (March 27th 2026, nanobanana.org)).

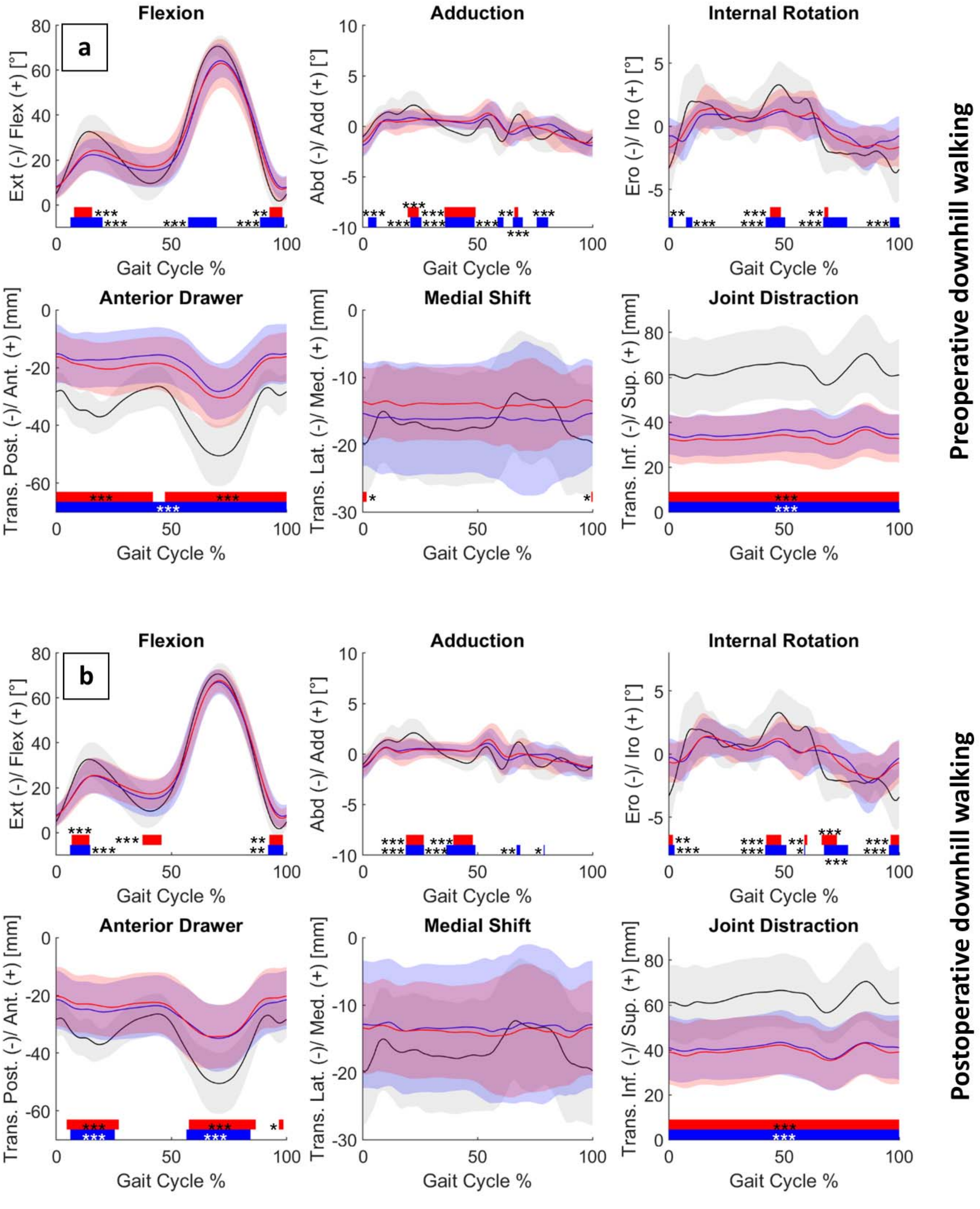

*Supplementary Figure S2: Mean ± SD (shaded areas) of pre- (a) and postoperative (b) level walking for MA (red), KA (blue), and control (black) groups. Joint rotations are shown in the upper row and translations in the lower row. Bars indicate significant differences between groups. Red: MA vs. C, blue: KA vs. C (*p = 0.02; **p = 0.01; ***p < 0.01).*

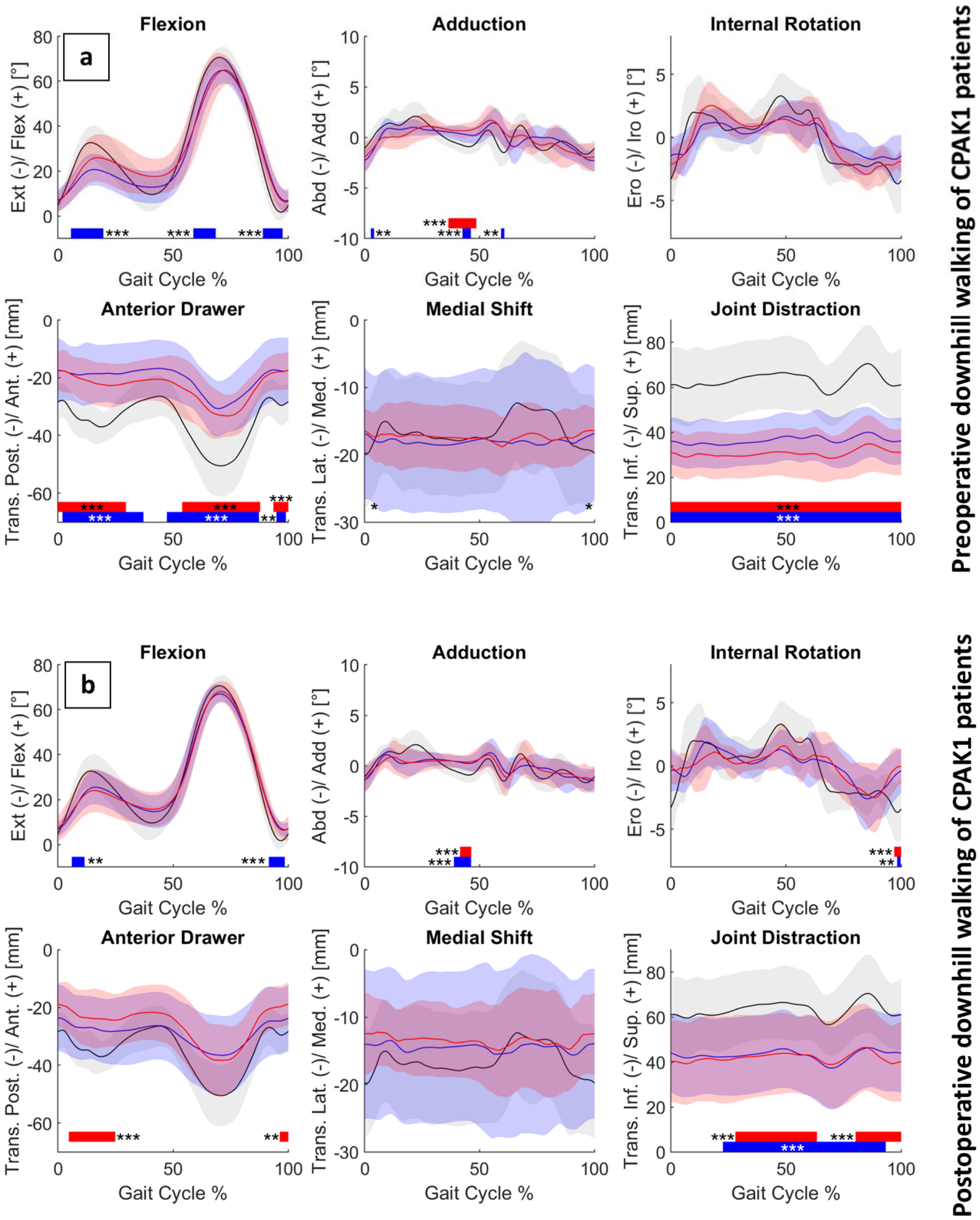

*Supplementary Figure S3: Mean ± SD (shaded areas) of pre- (a) and postoperative (b) downhill walking for MA (red), KA (blue), and control (black) groups. Joint rotations are shown in the upper row and translations in the lower row. Bar sindicate significant differences between groups. Red: MA vs. C, blue: KA vs. C (*p = 0.02; **p = 0.01; ***p < 0.01).*

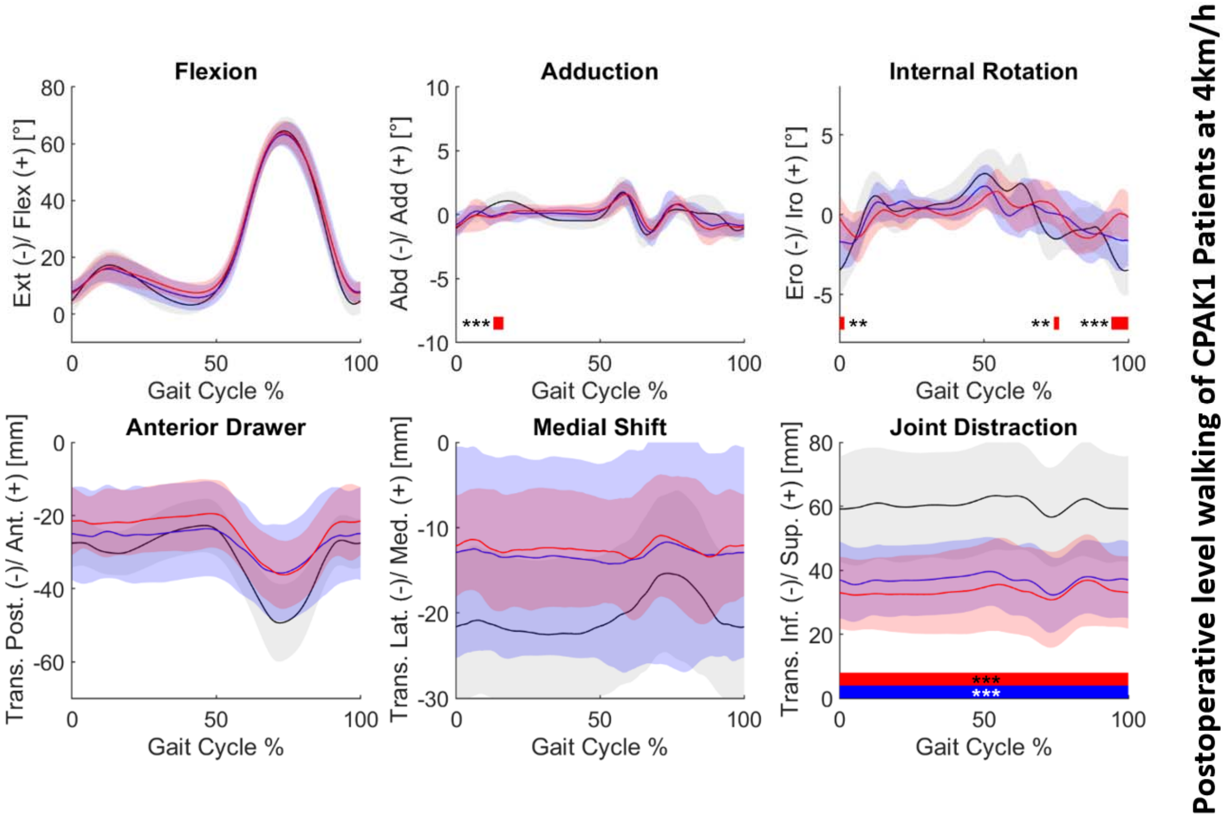

*Supplementary Figure S4: Mean ± SD (shaded areas) of postoperative (b) level walking for CPAK1 MA (red), CPAK1 KA (blue), and control (black) groups at 4 km/h. Joint rotations are shown in the upper row and translations in the lower row. Bars indicate significant differences between groups. Red: MA vs. C, blue: KA vs. C (*p = 0.02; **p = 0.01; ***p <0.01).*
