# Supplementary figures and images for: Six-degree-of-freedom knee motion during treadmill walking in mechanically and kinematically aligned TKA
Source: Sci Rep. 2026 May 14;16:15109. doi: 10.1038/s41598-026-52076-8 (PMC13172103; doi:10.1038/s41598-026-52076-8)

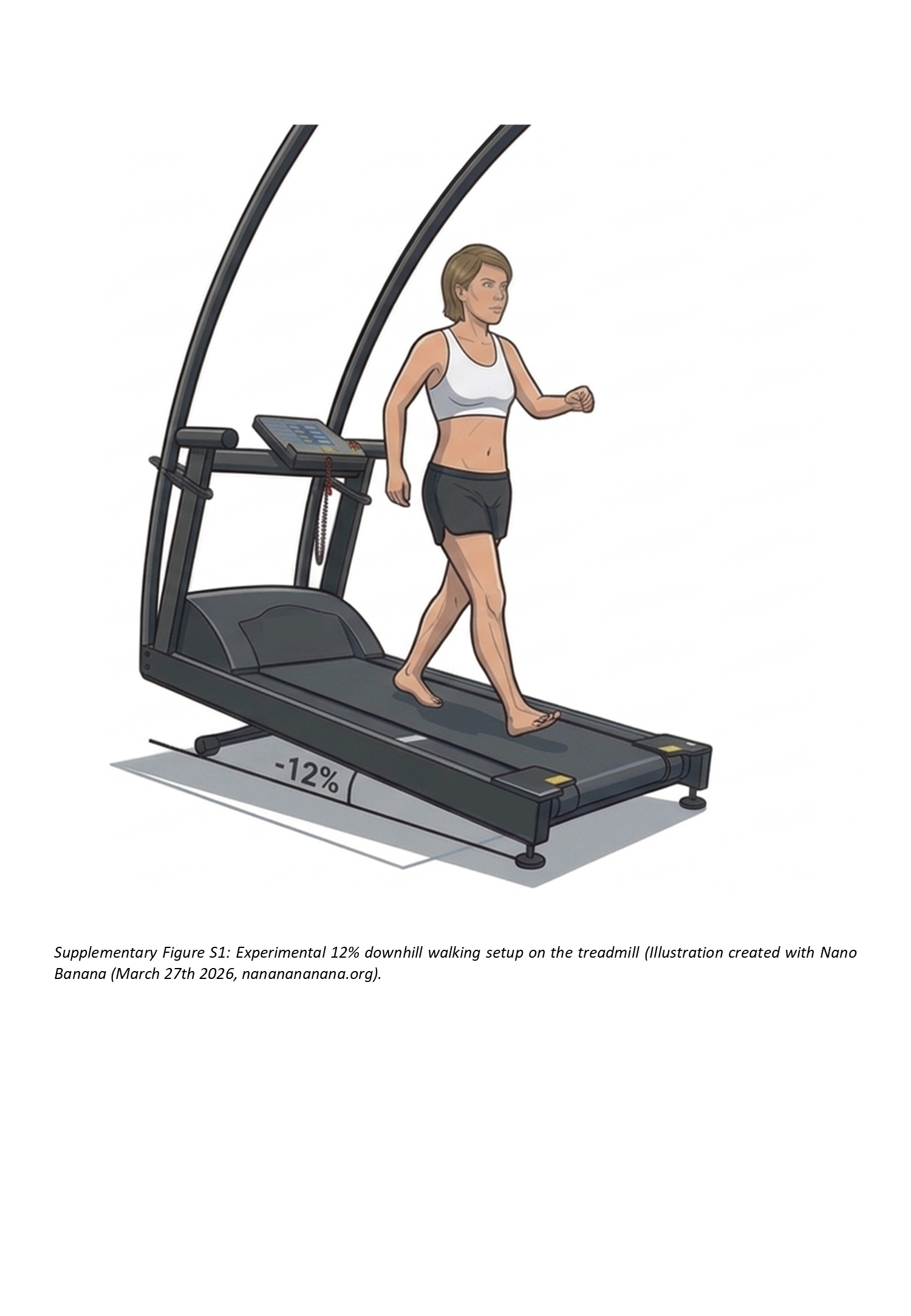

Supplement: Supplementary file 2 — Supplementary Material 2 [file 41598_2026_52076_MOESM2_ESM.png]
